# Multiple Analytical Approaches Reveal Distinct Gene-Environment Interactions in Smokers and Non Smokers in Lung Cancer

**DOI:** 10.1371/journal.pone.0029431

**Published:** 2011-12-19

**Authors:** Rakhshan Ihsan, Pradeep Singh Chauhan, Ashwani Kumar Mishra, Dhirendra Singh Yadav, Mishi Kaushal, Jagannath Dev Sharma, Eric Zomawia, Yogesh Verma, Sujala Kapur, Sunita Saxena

**Affiliations:** 1 National Institute of Pathology (Indian Council of Medical Research) Safdarjung Hospital Campus, New Delhi, India; 2 Dr. Bhubaneswar Borooah Cancer Institute, Gopi Nath Nagar, Guwahati, Assam, India; 3 Civil Hospital Aizawl, Mizoram, India; 4 Sir Thutob Namgyal Memorial Hospital, Gangtok, Sikkim, India; Oklahoma Medical Research Foundation, United States of America

## Abstract

Complex disease such as cancer results from interactions of multiple genetic and environmental factors. Studying these factors singularly cannot explain the underlying pathogenetic mechanism of the disease. Multi-analytical approach, including logistic regression (LR), classification and regression tree (CART) and multifactor dimensionality reduction (MDR), was applied in 188 lung cancer cases and 290 controls to explore high order interactions among xenobiotic metabolizing genes and environmental risk factors. Smoking was identified as the predominant risk factor by all three analytical approaches. Individually, CYP1A1*2A polymorphism was significantly associated with increased lung cancer risk (OR = 1.69;95%CI = 1.11–2.59,p = 0.01), whereas *EPHX1* Tyr113His and *SULT1A1* Arg213His conferred reduced risk (OR = 0.40;95%CI = 0.25–0.65,p<0.001 and OR = 0.51;95%CI = 0.33–0.78,p = 0.002 respectively). In smokers, *EPHX1* Tyr113His and *SULT1A1* Arg213His polymorphisms reduced the risk of lung cancer, whereas CYP1A1*2A, CYP1A1*2C and *GSTP1* Ile105Val imparted increased risk in non-smokers only. While exploring non-linear interactions through CART analysis, smokers carrying the combination of *EPHX1* 113TC (Tyr/His), *SULT1A1* 213GG (Arg/Arg) or AA (His/His) and *GSTM1* null genotypes showed the highest risk for lung cancer (OR = 3.73;95%CI = 1.33–10.55,p = 0.006), whereas combined effect of CYP1A1*2A 6235CC or TC, *SULT1A1* 213GG (Arg/Arg) and betel quid chewing showed maximum risk in non-smokers (OR = 2.93;95%CI = 1.15–7.51,p = 0.01). MDR analysis identified two distinct predictor models for the risk of lung cancer in smokers (tobacco chewing, *EPHX1* Tyr113His, and *SULT1A1* Arg213His) and non-smokers (CYP1A1*2A, *GSTP1* Ile105Val and *SULT1A1* Arg213His) with testing balance accuracy (TBA) of 0.6436 and 0.6677 respectively. Interaction entropy interpretations of MDR results showed non-additive interactions of tobacco chewing with *SULT1A1* Arg213His and *EPHX1* Tyr113His in smokers and *SULT1A1* Arg213His with *GSTP1* Ile105Val and CYP1A1*2C in nonsmokers. These results identified distinct gene-gene and gene environment interactions in smokers and non-smokers, which confirms the importance of multifactorial interaction in risk assessment of lung cancer.

## Introduction

Lung cancer is the most commonly diagnosed cancer and the leading cause of cancer death globally [1]. In India it constitutes 6.2% of all cancers with approximately 58,000 incident cases reported in 2008 and is the most frequent cancer in males [2]. North eastern (NE) part of India is showing a steady rise in cancer incidences and lung cancer is among the ten leading sites, with the highest age-adjusted incidence rate (AAR) in Mizoram state (24.5 in males and 26.3 in females). Aizwal district alone shows an AAR of 36.0 in males and 38.7 in females which is almost three to ten times higher than Delhi [3]. Incidence of lung cancer is also high among males in Silchar and Imphal districts. High incidence rates suggest role of both genetic as well as environmental factors such as smoking, tobacco use and dietary carcinogen consumption.

Individuals possessing modified ability to metabolize carcinogens such as polycyclic aromatic hydrocarbons (PAH), which are ubiquitous environmental, dietary, and tobacco carcinogens are at increased risk of developing cancer. Thus genetic variants in xenobiotic metabolizing genes can influence their clearance from circulation and determine response to such carcinogens. The phase I xenobiotic metabolizing enzymes like cytochrome P-450s (*CYP*s), alcohol dehydrogenase (*ALDH*) and epoxide hydroxylase (*EPHX*) usually activate the procarcinogens through oxidation and dehydrogenation thereby converting them into reactive metabolites. Phase II metabolic enzymes such as glutathione S-transferases (*GST*), sulfotransferase (*SULT*) and N-acetyltransferase (*NAT*) generally result in inactivation or detoxification of these reactive metabolites. Equilibrium between expression and activity levels of these xenobiotic-metabolizing enzymes of both phase I and II determine the relative level of detoxification of carcinogens. However, these pathways are also known to activate toxic and carcinogenic chemicals to electrophilic forms that react irreversibly with macromolecules such as proteins and nucleic acids leading to carcinogenesis.

Single nucleotide polymorphisms (SNPs) in xenobiotic metabolizing genes have been studied extensively with risk of lung cancer. A majority of these molecular epidemiological studies consider only the main effects of these SNPs and their observed strength of associations could be challenged by penetrance of the genetic variant. Furthermore, a single locus cannot account for genetic susceptibility in a complex disease such as cancer which involves multiple genetic variations and gene-environment interactions. Current evidences suggest that high order interactions in multigenic approach allow more precise delineation of risk groups [4], [5].

In the present study, two data mining approaches, CART and MDR were applied along with LR to detect high order gene-gene and gene environment interactions. Both CART and MDR assume model free and non-parametric methods of estimating non-linear interactions with low false-positives even on relatively small sample sizes. Model validation through permutation testing and false positive report probabilities were also done to overcome inaccurate estimation. Interaction entropy graphs were constructed to interpret combination effects identified by MDR. To further analyze possible effects of the *EPHX1* and *CYP1A1* SNPs, we estimated their haplotype frequencies and risk imparted towards lung cancer.

## Materials and Methods

### Study subjects

This study consisted of 188 histopathologically diagnosed lung cancer cases registered at Dr. Bhubaneswar Borooah Cancer Institute, Guwahati, Civil Hospital, Aizawl, and Sir Thutob Namgyal Memorial Hospital, Gangtok, the collaborating centers in north east India. Incident cases during the period of December 2006 to 2009 and willing to participate in the study were included. 290 voluntary, age (±5 years) and sex matched individuals were selected from the unrelated attendants who accompanied cancer patients. This provided a readily available and cooperative source of controls from the same socio-economic background as the cases reducing confounding biases. As our collaborating centers were public hospitals a large majority of subjects belonged to lower to middle socio-economic background. Demographic data and characteristics such as age, sex, smoking habit, usage of tobacco, betel quid and alcohol, were obtained from subjects in a standard questionnaire used for all the centers, in an in-person interview by a trained data collector. A majority of cases and controls were literate with full primary schooling and some upto the college level. The occupational history of the study participants revealed that most of them were farm laborers or engaged in petty jobs and the nature of such jobs did not exposed them to any occupational hazards. Any history of past or present illness was enquired or if undergoing any medication at the time of enrolment. Patients with only lung as their primary site of cancer were included. Any subject with history of familial malignancy or pulmonary infectious disease was excluded both from case and control. Final selected controls were included on the basis of no history of any obvious disease and those not taking any medication at the time of recruitment. All subjects provided written informed consent for participation in this research which was done under a protocol approved by the institutional ethics committee of Regional Medical Research Centre, North East Region (Indian Council of Medical Research). Smokers, chewers and drinkers were classified into two categories ever and never. For smoking, an individual who had never smoked or smoked less than 100 cigarettes in their lifetime and were not smoking at the time of reporting was considered never smoker or non-smokers. Ever smokers or smokers category included current smokers, and those who had quit within <1 year of reporting [6]. 5 ml of blood was collected in EDTA vials and stored under -70°C until processed.

### Genotyping

Genomic DNA was isolated using Qiagen Blood DNA Isolation kit (Qiagen GmbH, Germany) and stored at −30°C till further analysis. Details for SNPs selected for the study are summarized in Table S1. The deletion variants in *GSTM1* and *GSTT1* were determined by multiplex polymerase chain reaction protocol and SNPs in *CYP1A1*, *EPHX1, GSTP1, SULT1A1* were determined by polymerase chain reaction–restriction fragment length polymorphism assays as previously described [7]–[12]. 10% of the randomly selected cases and controls were genotyped twice for each SNP, however no discrepancies were observed.

### Statistical Analysis

Cases were individually matched with controls on the basis of age (±5 years), sex and ethnicity, in a ratio of approximately 1∶1.5. Difference in the distribution of demographic characteristics and genotype frequencies between cases and controls were evaluated using the Chi Square (χ^2^) and Fisher's Exact test wherever appropriate. Hardy–Weinberg equilibrium (HWE) was assessed by using the χ^2^-test. Estimates of risk to cancer, imparted by genotypes and other covariates as tobacco smoking, tobacco chewing, betel quid chewing and alcohol consumption were determined by deriving the odds ratio (OR) and its corresponding 95% confidence interval (95% CIs) using multivariable conditional logistic regression. For all the tests a two sided p<0.05 was considered statistically significant. The data analysis was performed on the Intercooled Stata 8.0 statistical software package (Stata Co., College Station, TX).

### Haplotype Analysis

Haplotypes were constructed from the unphased diploid genotype data using the Expectation Maximization-based algorithm. Individual haplotypes and their estimated population frequencies were inferred and estimates of linkage disequilibrium (D') between SNPs were calculated using Haploview software ver.4.1.

### Identification of High Order Interactions

High order interactions were determined using CART, MDR and interaction entropy graphs.

#### CART

A binary recursive partitioning method was used to produce a decision tree that identified specific combinations of contributing factors associated with lung cancer risk using the commercially available CART software (version 6.6, Salford Systems) [13]. Tree splitting was done till terminal nodes reached a pre- specified minimum size of 10 subjects. Optimal tree was selected using one standard error (1-SE) rule and 10 fold cross validation. Subgroups of individuals with differential risk patterns were identified in the different order of nodes, indicating the presence of gene-gene and gene-environment interactions. Fischer's Exact test was used to calculate relative risk in each terminal node of the tree.

#### MDR

The MDR software was developed by Ritchie et. al. in 2001 [4] and reviewed by Moore et al [14]. Genotype and environmental factors were pooled into high and low risk group, effectively reducing the multifactor prediction from n dimension to one dimension using MDR software (version 2.0 beta) (http://www.epistasis.org). We applied Tuned ReliefF (TuRF) filter algorithm to remove noisy SNPs and avoid overfitting of data. Best models for each locus were selected by repeating the analysis for up to 10 seeds and applying 10 folds cross validation each time. Statistical significance of the best models selected for each locus was determined using 1000 fold permutation testing. p-values hence obtained for TBA and cross validation consistency (CVC), were considered statistically significant at 0.05 levels.

### False Positive Report Probability (FPRP)

Reports of gene-environment interaction studies are often challenged by false positive discoveries especially when results are generated by multiple comparisons. To estimate the FPRP and to evaluate robustness of the findings from MDR analysis we used the Bayesian approach described by Wacholder et. al. [15]. The method requires prior probabilities that the genetic variant and disease association is real. As prior probability can be a subjective measure and can be influenced by several factors, usually a wide range is reported by studies. Considering poor epidemiological data from the study population and inconsistent association of the SNPs with lung cancer risk we set a fairly wider range of prior probabilities (10^−6^ to 10^−1^) with an estimated statistical power to detect an OR of 1.5 and 2.0 and α level equal to the observed p-value. The FPRP cutoff point was stringently kept to 0.2.

### Interaction entropy graphs

Interaction graphs were built to visualize and interpret the results obtained from MDR using Orange machine learning software package [16]. Interaction graphs use entropy estimates as described by Jakulin et al. [17] for determining the gain in information about a class variable (e.g. case–control status) from merging two variables together over that provided by the variables independently. This measure of entropy is useful for building interaction graphs that facilitate the interpretation of the relationship between variables. Interaction graphs are comprised of a node for each variable with pairwise connections between them. The percentage of entropy removed (i.e. information gain) by each variable is visualized for each node. The percentage of entropy removed for each pairwise Cartesian product of variables was visualized for each connection. Thus, the independent main effects of each SNP can be compared to the interaction effect. Positive entropy (plotted in green) indicates non-linear interaction while negative entropy (plotted in red) indicates redundancy. Entropy value equal to zero indicates independence or a mixture of synergy and redundancy.

## Results

### Characteristics of study subjects

The distribution of gender and ethnicity was similar for cases and controls. The frequency distribution of males and females were 77.1% and 22.9% in cases and 76.2% and 23.85 in controls respectively. Mean age of cases and controls was 60.41±10.58 (range 30–82 yrs) and 57.19±10.75 (range 32–85 yrs) respectively. The distribution of all SNPs in control was in agreement with HWE (p>0.05), however alleles of *EPHX1* Tyr113His and *SULT1A1* Arg213His polymorphisms in cases did not follow HWE (p<0.001 and p = 0.004 respectively).

### Association of genetic and environmental factors with lung cancer risk by LR analysis

The distribution and main effects of genetic and environmental factors is summarized in Table 1. Risk habits such as smoking, tobacco chewing and betel quid chewing were predominant among cases. However only smoking and betel quid chewing were significantly associated with increased risk for lung cancer (OR = 3.06;95%CI = 1.94−4.83;p<0.001 and OR = 1.86; 95%CI = 1.21−2.84;p = 0.004 respectively). Genotype distribution of CYP1A1*2A, *EPHX1* Tyr113His, *SULT1A1* Arg213His and *GSTT1* null polymorphism were significantly different in cases from controls (p = 0.014, p<0.001, p = 0.01 and p = 0.04 respectively). Main effects of genotypes in lung cancer susceptibility were evaluated using conditional multivariable logistic regression. Heterozygous genotype in CYP1A1*2A was associated with increased risk (OR = 1.69,95% CI = 1.11−2.59; p = 0.01) whereas heterozygous genotypes in *EPHX1* Tyr113His and *SULT1A1* Arg213His imparted reduced risk towards lung cancer (OR = 0.40;95%C.I = 0.25−0.65,p<0.001 and OR = 0.51;p = 0.33x−0.78,p = 0.002 respectively). CYP1A1*2A and *EPHX1* His139Arg polymorphisms were associated with increased risk to lung cancer in dominant genetic model, whereas *EPHX1* Tyr113His and *SULT1A1* Arg213His imparted reduced risk in recessive genetic model (Table S2).

**Table 1:** Association of genotypes of xenobiotic metabolizing genes and environmental risk factors with lung cancer susceptibility.

FACTORS	CATEGORIES	GENOTYPE	CASES		CONTROLS		OR (95% C.I.)	p value
Genetic Factors#			n	%	n	%		
***CYP1A1*** * *2A* a	TT	T6235T	55	29.3	122	42.1	1..0	
	TC	T6235C	103	54.8	124	42.8	1.69 (1.11–2.59)	0.01
	CC	C6235C	30	16.0	44	15.2	1.53 (0.84–2.78)	0.15
***CYP1A1*** * *2C*	AA	Ile462Ile	122	64.9	206	71.0	1..0	
	AG	Ile462Val	56	29.8	77	26.6	1.16 (0.75–1.80)	0.48
	GG	Val462Val	10	5.3	7	2.4	2.18 (0.78–6.09)	0.13
***EPHX1*** Tyr113His a	TT	Tyr113Tyr	82	43.6	94	32.4	1.0	
	TC	Tyr113His	51	27.1	133	45.9	0.40 (0.25–0.65)	<0.001
	CC	His113His	55	29.3	63	21.7	1.00 (0.60–1.67)	0.98
***EPHX1*** His139Arg	AA	His139His	121	64.4	212	73.1	1.0	
	AG	His139Arg	59	31.4	70	24.1	1.45 (0.92–2.27)	0.10
	GG	Arg139Arg	8	4.3	8	2.8	2.41 (0.79–7.36)	0.12
***GSTM1***	Wild Type	Present	122	64.9	177	61.0	1.0	
	Null	Null Genotype	66	35.1	113	39.0	0.95 (0.63–1.41)	0.80
***GSTT1*** a	Wild Type	Present	155	82.4	217	74.8	1.0	
	Null	Null Genotype	33	17.6	73	25.2	0.62 (0.38–1.02)	0.06
***GSTP1***	AA	Ile105Ile	102	54.3	179	61.7	1.0	
	AG	Ile105Val	77	41.0	96	33.1	1.46 (0.95–2.23)	0.07
	GG	Val105Val	9	4.8	15	5.2	1.09 (0.43–2.77)	0.84
***SULT1A1*** a	GG	Arg213Arg	123	65.4	153	52.8	1.0	
	GA	Arg213His	50	26.6	116	40.0	0.51 (0.33–0.78)	0.002
	AA	His213His	15	8.0	21	7.2	0.87 (0.42–1.82)	0.72
**Environmental Factors##**								
**Smoking status^a^**	Non-smokers		56	29.8	151	52.1	1.0	
	Smokers		132	70.2	139	47.9	3.06 (1.94–4.83)	<0.001*
**Tobacco chewing^a^**	Non-chewers		92	48.9	172	59.3	1.0	
	Chewers		96	51.1	118	40.7	1.24 (0.82–1.85)	0.293
**Betel quid chewing^a^**	Non chewers		52	27.7	131	45.2	1.0	
	Chewers		136	72.3	159	54.8	1.86 (1.21–2.84)	0.004*
**Alcohol consumption**	Non-alcoholic		135	71.8	207	71.4	1.0	
	Alcoholic		53	28.2	83	28.6	0.87 (0.56–1.37)	0.57

a χ^2^ significant; p<0.05.

# ORs adjusted for all environmental factors.

## ORs adjusted for all genetic factors.

*Significant after p-value adjustment for multiple comparision (Sidak correction).

### Haplotype analysis

Table 2 summarizes the associations between the frequency distributions of the haplotypes in *CYP1A1* and *EPHX1* genes and the risk of lung cancer. The odds ratios were calculated using the most common haplotype as the reference group. In *CYP1A1*, “TA” haplotype was the most frequent among both cases and controls and showed significant association. Only *CYP1A1*-CG haplotype imparted increased risk to lung cancer (OR = 1.49;95%CI = 1.00−2.21,p = 0.04). In *EPHX1*, the “TA” haplotype was the most common with frequencies of 44.79% and 45.04% in cases and controls respectively. No haplotype was found to be significantly associated with lung cancer risk.

**Table 2:** Distribution of CYP1A1 and EPHX1 haplotype frequency among lung cancer cases and controls.

	HAPLOTYPE	CASE ( 376)		CONTROL ( 580)		χ^2^	P value	OR (95%CI)	P value	D'
		%	n	%	n					
CYP1A12A*2C	TA	53.34	201	60.80	352	5.00	0.02	1.00		0.72
	TG	3.31	12	2.65	16	0.21	0.64	1.31 (0.57–3.00)	0.50	
	CA	26.45	99	23.51	137	0.95	0.32	1.26 (0.91–1.74)	0.15	
	CG	16.90	64	13.04	75	2.94	0.08	1.49 (1.00–2.21)	0.04	
***EPHX1*** Tyr113His * His139Arg	TA	44.79	168	45.04	262	0.05	0.81	1.00		0.21
	TG	12.39	47	10.31	59	1.57	0.20	1.23(0.78–1.94)	0.30	
	CA	35.26	133	40.13	233	1.82	0.17	0.88 (0.65–1.19)	0.42	
	CG	7.56	28	4.52	26	2.64	0.10	1.67 (0.91–3.06)	0.07	

**D**' Linkage Disequilibrium.

### Risk associated with SNPs stratified by smoking

Since smoking is a well established risk factor to lung cancer and was the strongest independent risk factor in LR, we further stratified the data by smoking status. Distribution and risk associated with genetic factors after stratification is shown in Table 3. Heterozygous and homozygous variant genotypes of CYP1A1*2A polymorphism imparted significant risk in non-smokers (OR = 2.88;95%CI = 1.22−6.81,p = 0.016 and OR = 4.35;95%CI = 1.47−12.84,p = 0.008). Also, CYP1A1*2C variant genotype and *GSTP1* Ile105Val heterozygous genotype were significantly associated with increased risk in non-smokers (OR = 11.81;95%CI = 1.24−111.98,p = 0.03 and OR = 2.40;95%CI = 1.15−5.03,p = 0.01). Heterozygous genotypes in *EPHX1* Tyr113His and *SULT1A1* Arg213His were associated with 66% and 55% reduced risk in smokers (OR = 0.34;95%CI = 0.18−0.63,p = 0.001 and OR = 0.45;95%CI = 0.25−0.80,p = 0.007 respectively). However heterozygous genotype in *EPHX1* His139Arg conferred significant risk in smokers (OR = 1.92;95%CI = 1.07−3.45,p = 0.02).

**Table 3:** Main effects of genotypes on lung cancer risk stratified by smoking.

Polymorphism	Genotype	Smoker		Non Smoker	
		Case/Control (n,%)	OR (95% C.I.),p value*	Case/Control (n,%)	OR (95% C.I.),p value*
***CYP1A1*** * ***2A***	TT	44(33.3)/57(41.0)	1.0	11(19.6)/65(28.6)	1.0
	TC	74(56.1)/61(43.9)	1.45(0.84–2.50),0.17	29(51.8)/63(41.7)	2.88(1.22–6.81),0.016
	CC	14(10.6)/21(15.1)	0.83(0.36–1.91),0.66	16(28.6)/23(15.2)	4.35(1.47–12.84),0.008#
***CYP1A1*** * ***2C***	AA	86(65.2)/93(66.9)	1.0	36(64.3)/113(74.8)	1.0
	AG	40(30.3)/40(28.8)	1.14(0.65–2.02),0.63	16(28.6)/37(24.5)	1.53(0.67–3.48),0.30
	GG	6(4.5)/6(4.3)	1.71(0.43–6.74),0.43	4(7.1)/1(0.7)	11.81(1.24–111.98),0.03
***EPHX1 Tyr113His***	TT	60(45.5)/41(29.5)	1.0	22(39.3)/53(35.1)	1.0
	TC	35(26.5)/71(51.1)	0.34(0.18–0.63),0.001#	16(28.6)/62(41.1)	0.62(0.25–1.54),0.30
	CC	37(28.0)/27(19.4)	1.14(0.57–2.29),0.69	18(32.1)/36(23.8)	1.03(0.41–2.56),0.94
***EPHX1 His139Arg***	AA	80(60.6)/103(74.1)	1.0	41(73.2)/109(72.2)	1.0
	AG	48(36.4)/32(23.0)	1.92(1.07–3.45),0.02	11(19.6)/38(25.2)	0.98(0.41–2.36),0.98
	GG	4(3.0)/4(2.9)	1.39(0.31–6.25),0.66	4(7.1)/4(2.6)	4.25(0.54–33.15),0.16
***GSTM1***	WildType	91(68.9)/86(61.9)	1.0	31(55.4)/91(60.3)	1.0
	Null	41(31.1)/53(38.1)	0.87(0.51–1.48),0.62	25(44.6)/60(39.7)	1.25(0.61–2.54),0.53
***GSTT1***	WildType	106(80.3)/104(74.8)	1.0	49(87.5)/113(74.8)	1.0
	Null	26(19.7)/35(25.2)	0.75(0.40–1.41),0.37	7(12.5)/38(25.2)	0.48(0.19–1.20),0.11
***GSTP1***	AA	69(52.3)/77(55.4)	1.0	33(58.9)/102(67.5)	1.0
	AG	54(40.9)/55(39.6)	1.35(0.77–2.36),0.29	23(41.1)/41(27.2)	2.40(1.15–5.03),0.01#
	GG	9(6.8)/7(5.0)	1.49(0.49–4.56),0.47	0/8(5.3)	NA
***SULT1A1***	GG	84 (63.6)/69 (49.6)	1.0	39(69.6)/84(55.6)	1.0
	GA	35 (26.5)/58 (41.7)	0.45(0.25–0.80),0.007#	15(26.8)/58(38.4)	0.54(0.24–1.19),0.13
	AA	13 (9.8)/12 (8.6)	1.11(0.45–2.74),0.81	2(3.6)/9(6.0)	0.48(0.09–2.54),0.39

*p values adjusted for tobacco chewing, betel quid chewing and alcohol consumption.

# Significant after p-value adjustment for multiple comparision (Sidak correction).

### CART analysis

**Figure 1:**
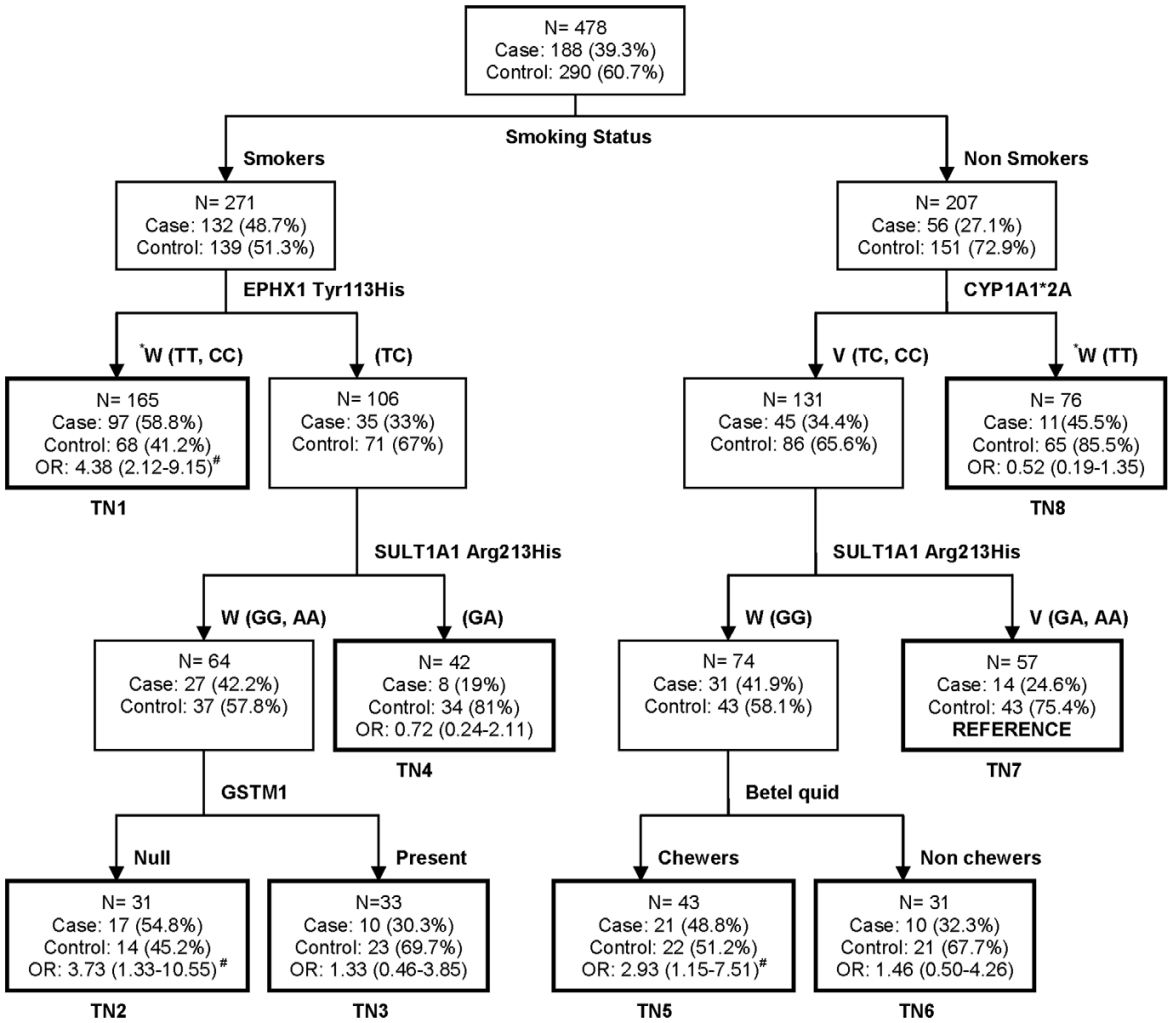
Classification and regression tree model for xenobiotic metabolizing gene polymorphisms and environmental risk factors. Terminal nodes are thick bordered. ^*^W: Wild type genotype; V: Variant genotype, TN: Terminal Node, ^#^p value <0.05

Figure 1 shows the selected CART model constructed on all investigated genetic variants and environmental risk factors. The final tree contained eight terminal nodes. The first split of the root node was on smoking habit, indicating that smoking is the strongest risk factor for lung cancer. Among smokers, the subsequent splits showed interactions between *EPHX1* Tyr113His, *SULT1A1* Arg213His and *GSTM1*. In non-smokers first split was on CYP1A1*2A status, which was in concordance with the LR analysis where CYP1A1*2A showed strong association to risk only in nonsmokers. Further interactions were predicted by *SULT1A1* Arg213His polymorphism and betel quid status. Terminal node 7, which comprised of least percentage of cases in non-smokers, was taken as reference to calculate OR for other terminal nodes. Among smokers maximum risk was observed for terminal node1 consisting of *EPHX1* 113TT (Tyr/Tyr) or -113CC (His/His) genotypes (OR = 4.38;95%CI = 2.12−9.15) and for terminal node 2 with combination of *EPHX1* 113TC (Tyr/His), *SULT1A1* 213GG (Arg/Arg) or AA (His/His) and *GSTM1* null genotypes (OR =  3.73;95%CI = 1.33−10.55, p = 0.006). In non-smokers high risk was seen for terminal node 5 comprising of CYP1A1*2A 6235CC or TC, *SULT1A1* 213GG (Arg/Arg) and betel quid chewing (OR = 2.93;95%CI = 1.15−7.51, p =  0.01). Parallel to the above, CART analysis on separate data sets of smokers and non-smokers was also performed. However, we did not detect any high-order interaction in these analyses (data not shown).

### MDR Analysis

MDR analysis was applied to further explore gene-gene and gene-environment interactions. Best predictive models up to 4 orders of interaction, along with their CVC and TBA are summarized in Table 4. The analysis was run separately for total data set and data sets stratified on smoking status. For total data set, smoking was the best one locus model with highest CVC (10/10) and testing accuracy of 0.6114 which was statistically significant (p<0.001) determined by 1000 fold permutation testing. For a 2-locus interaction, combination of smoking and *EPHX1* Tyr113His was most significant with CVC of 10/10 and TBA of 0.6407 (p<0.001). The 3 locus model consisted of smoking, *EPHX1* Tyr113His and *EPHX1* His139Arg with TBA of 0.6497 (p<0.001) and CVC of 10/10. The 4 loci interaction model of smoking, *EPHX1* Tyr113His, *EPHX1* His139Arg and *SULT1A1* Arg213His, was the best model identified, with maximum CVC (10/10) and TBA (0.6503, p<0.001). This model had a chi-square value of 66.31 (p<0.0001) and an OR of 4.93 (95%CI = 3.32−7.33). In smokers the best interaction model was the three loci model consisting of tobacco chewing, *EPHX1* Tyr113His and *SULT1A1* Arg213His having maximum CVC (10/10) and TBA (0.6436, p<0.001) among all models identified. The model imparted 3.5 fold increased risk for lung cancer (95%CI = 2.69−7.69). In non-smokers the best model was the three loci model comprising of CYP1A1*2A, *GSTP1* Ile105Val and *SULT1A1* Arg213His with CVC of 10/10 and TBA of 0.6677 (p<0.005) and an OR of 7.32 (95%CI = 3.24−16.53).

**Table 4:** Results of MDR analysis.

	No. of Locus	Model	p value (χ^2^ test)	TBA	p-value*	CVC	p-value*
**Total Data Set**							
	1st order	Smk	p < 0.0001	0.6114	<0.001	10	0.391
	2^nd^ order	Smk Ex3	p < 0.0001	0.6407	<0.001	10	0.391
	3^rd^ order	Smk Ex3 Ex4	p < 0.0001	0.6497	<0.001	10	0.391
	4^th^ order**	**Smk Ex3 Ex4 SULT**	**p < 0.0001**	**0.6503**	**<0.001**	**10**	**0.391**
**Smokers**							
	1st order	Ex3	p < 0.0001	0.6228	0.012	10	0.402
	2^nd^ order	Tbc Ex3	p < 0.0001	0.6105	<0.02	9	0.623
	3^rd^ order**	**Tbc Ex3 SULT**	**p < 0.0001**	**0.6436**	**<0.001**	**10**	**0.402**
	4^th^ order	Tbc Alc Ex3 SULT	p < 0.0001	0.6268	<0.008	7	0.846
**Non Smokers**							
	1st order	2A	p = 0.0019	0.6170	0.09	10	0.372
	2^nd^ order	2A SULT	p = 0.0004	0.5562	0.46	8	0.734
	3^rd^ order**	**2A P1 SULT**	**p < 0.0001**	**0.6677**	**<0.005**	**10**	**0.372**
	4^th^ order	2A 2C P1 SULT	p < 0.0001	0.6439	<0.021	10	0.372

*1,000-fold permutation test. **Best models selected with maximum cross-validation consistency **(CVC)** and maximum testing balance accuracy **(TBA)**. **Labels:** Smk: smoking, Ex3: *EPHX1 Tyr113His*, Ex4: *EPHX1 His139Arg*, SULT: *SULT1A1 Arg213His*, Tbc: tobacco chewing, Alc: alcohol consumption, 2A: CYP1A1*2A, P1: *GSTP1 Ile105Val*, 2C: CYP1A1*2C.

### False positive report probability (FPRP)

Table 5 shows the FPRPs for the 3 best models obtained from MDR analysis. The 4-loci predictor model on total data set and 3-loci model in smokers showed excellent reliability even when assuming very low prior probabilities (from 10^−3^ to 10^−6^) for detecting ORs of 1.5 and 2.0. However the best model selected in non smoker category showed true association only at high probability of 10^−1^ for detecting OR = 1.5 and till 10^−2^ for detecting OR = 2.0.

**Table 5:** False positive report probability and odds ratio for best model of MDR analysis.

	OR (95% CI) p value	OR = 1.5	Prior Probability						OR = 2.0	Prior Probability					
		Power	10^−1^	10^−2^	10^−3^	10^−4^	10^−5^	10^−6^	Power	10^−1^	10^−2^	10^−3^	10^−4^	10^−5^	10^−6^
**Total Data Set Smk Ex3 Ex4 SULT**	4.93 (3.32–7.33) p < 0.0001	0.0001	**<10^−4^**	**<10^−4^**	**0.001**	**0.015**	**0.131**	**0.131**	0.0001	**<10^−4^**	**<10^−4^**	**<10^−4^**	**<10^−4^**	**<10^−4^**	**<10^−4^**
**Smokers Tbc Ex3 SULT**	4.55 (2.69–7.69) p < 0.0001	0.0001	**0.008**	**0.081**	0.472	0.900	0.989	0.989	0.001	**<10^−4^**	**0.001**	**0.014**	**0.125**	0.588	0.588
**Non Smokers 2A P1 SULT**	7.32 (3.24–6.53) p < 0.0001	0.0001	**0.180**	0.708	0.961	0.996	1.00	1.00	0.001	**0.016**	**0.155**	0.650	0.949	0.995	0.995

Prior probabilities ranging from 0.1 to 10^−6^, with the estimated statistical power to detect an OR of 1.5 or 2.0 with α level equal to the observed p-value.

**Bold type** indicates the FPRP for the most likely prior probabilities i.e. a noteworthy association at the 0.2 FPRP.

### Interaction entropy graphs

**Figure 2:**
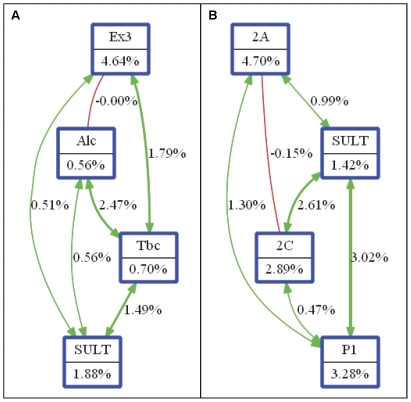
Interaction entropy graphs. The interaction model describes the percentage of the entropy (information gain) removed by each variable (main effect: represented by nodes) and by each pairwise combination of attributes (interaction effect: represented by connections). Attributes are selected on the basis of MDR results obtained in case of (**A**) Smokers and (**B**) Non smokers. **Labels:** Ex3: *EPHX1* Tyr113His, Alc: alcohol consumption, Tbc: Tobacco chewing, SULT: *SULT1A1* Arg213His, 2A: CYP1A1*2A, 2C: CYP1A1*2C, P1: *GSTP1* Ile105Val.

After identifying the high-risk combinations using MDR approach, interaction entropy algorithm was applied to interpret relationship between the variables. Graphs were constructed on MDR results obtained from analysis on total data set (Figure S1) and on data set stratified by smoking (Figure 2). In smokers, *EPHX1* Tyr113His had a large independent effect (4.64%) and a non-additive interaction with tobacco chewing (entropy 1.79%). Considerable entropy was associated with *SULT1A1* Arg213His (1.88%) and its interaction with tobacco chewing further removed 1.49% of entropy from case-control group. However we did not detect any non-linear interaction between the two SNPs in the model. We found small percentages of the entropy in case–control status explained by alcohol consumption (0.56%) and tobacco chewing (0.70%) independently, but a large percentage of entropy explained by the interaction between these two environmental factors (2.47%). In non-smokers, CYP1A1*2A showed strongest main effect with entropy removal of 4.7%. *GSTP1* Ile105Val too had a strong independent effect (entropy removal = 3.28%) and its interaction with *SULT1A1* Arg213His further removed 3.02% of entropy. A strong synergistic interaction was observed between *SULT1A1* Arg213His and CYP1A1*2C as the combination removed an additional 2.61% of the total entropy.

## Discussion

The present study used multiple analytical methods to first assess associations and then explore possible interactions of xenobiotic metabolizing genes with environmental factors in risk to lung cancer. The applied data mining approaches have the ability to search and identify interactions regardless of the significance of the main effects. The most significant finding of this study is the consistently identified gene-gene and gene environment interactions by all the three statistical approaches.

Smoking is the primary etiological factor in lung cancer. The same was reflected in the present study as smoking showed strong association in LR, best one factor model in MDR and formed first split in CART. Interaction of *EPHX1* Tyr113His and *SULT1A1* Arg213His was consistently identified in smokers. Both *EPHX1* Tyr113His and *SULT1A1* Arg213His conferred reduced risk in smoker subset in LR. The two polymorphisms along with *EPHX1* His139Arg formed the best predictor model in MDR analysis in smokers and also formed subsequent splits within smokers in CART. EPHX1 enzyme catabolizes epoxides from PAH into dihydrodiols, which involves generation of more reactive carcinogenic metabolites. Substitution of a variant His allele at codon 113 (*EPHX1* Tyr113His) decreases the activity of this enzyme [18] thereby reduces the risk of cancer. Studies on lung cancer suggest protective effect for *His*113 (slow type) as compared to *Tyr*113 (fast type) which imparts increased lung caner risk [19]–[21]. The variant allele has also been suggested to decrease the risk of ovarian cancer [22]. We have earlier reported similar results from the same population in esophageal cancer showing *His*113 allele to be associated with a significantly reduced risk in smokers [23]. Reflecting the same, in CART analysis Terminal node 1 of imparts over 4 fold high risk to smokers possibly due high proportion of the wild *Tyr*113 homozygous genotype. Sulphonation reaction of *SULT1A1* is a detoxification reaction, however it also involves bioactivation of certain procarcinogens, including heterocyclic amines and PAHs to form carcinogen-DNA adduct [24], [25]. In vitro model studies suggest that substitution of histidine at position 213 in the amino acid sequence is associated with decreased substrate affinity and a lower level of protein [26] which might protect against chemical carcinogenesis of PAHs in lung cancer [27]. Results on association of *SULT1A1* Arg213His and risk of cancer are inconsistent, from null association with risk of colorectal cancer [28] and prostate cancer [29] to increase in risk of breast cancer associated with *His*213 allele [30]. Another study on colorectal cancer showed a significantly reduced risk for individuals carrying *His*213 allele [31]. A Meta-analysis by Kotnis et al [32] showed a significant protective effect of the polymorphism in seven studies of genitourinary cancers.

Among non-smokers CYP1A1*2A and *GSTP1* Ile105Val were the most important polymorphisms identified for lung cancer development. The variant allele of both the polymorphisms conferred significant risk in the non smoking subgroup in LR analysis. Similarly, MDR 3 loci model of CYP1A1*2A, *GSTP1* Ile105Val and *SULT1A1Arg213His* polymorphisms was the best predictor of risk in non-smokers. The *CYP1A1* 6235T>C MspI (CYP1A1*2A) polymorphism, is associated with higher enzymatic activity towards benzopyrene [33], [34]. Investigations on association between *CYP1A1* polymorphisms and lung cancer have yielded equivocal results [35], [36]. Similar to our findings, a study by Taioli et. al. [37] reported association of CYP1A1*2A variant allele with lung cancer, however after stratification by smoking the association remained confined to non-smokers only. Further, in a pooled analysis of 11 studies on CYP1A1*2C polymorphism in lung cancer, Le Marchand et al [38] found it to be associated with risk in non-smokers, a finding which corroborates our results. Another study by Jose et al [39] on lung cancer found no association of any *CYP1A1* polymorphism with smokers. Similar results were reported in colorectal cancer where heterozygous and variant genotypes of both CYP1A1*2A and CYP1A1*2C conferred risk in combinations with NAT2 only among non-smokers [40]. *In vitro* cDNA expression study suggests that *GSTP1* with 105Val variant results in a protein with reduced enzyme activity [41], however it is reported to play an unlikely role for smoking-related cancers [42]. Similar observation has been reported from breast cancer [10]. Probably the precise role of *GSTP1* in carcinogenesis can be determined by the kind of xenobiotic involved owing to its substrate specificity and affinity [43].

Confirming to its exploratory nature, CART analysis identified two more risk factors, *GSTM1* null genotype in smokers and betel quid chewing in non-smokers. The results are quite plausible because both hold functional and biological significance. High risk for smoking related lung cancer has been reported in individuals deficient in *GSTM1* [44]–[46]. Smokers with the *GSTM1* enzyme have approximately one-third of the risk for lung carcinoma than smokers without the enzyme [47]. There are numerous reports of association between *GSTM1* null genotype and smoking in various cancers including esophageal [48], bladder [49] colorectal [50] and oral [51]. A recent study by Wen et. al. [52] showed betel quid chewing increases lung cancer risk in non-smokers, with smoking habit further enhancing the risk. Betel quid chewing is a unique and widespread habit in the north-eastern (NE) region of India. Betel quid is a chewing mixture of whole betel/areca nut wrapped with betel leaves spread with white lime with frequent addition of tobacco. It is known to contain phenolic compounds and alkaloids, besides nitrosamines are formed from an in vivo reaction of betel arecoline, nitrite and thiocynate, all of which act as carcinogens [53]. Studies have reported association between betel quid chewing and cancer risk. Significant association of betel quid chewing with risk of oral, stomach [54] esophageal [23] and breast cancer [55] has been reported from the study population. It would be reasonable to assume that the association of betel quid chewing with lung cancer is a result of a complex combination of direct and indirect action of tobacco carcinogens contained in it.

A post-hoc analysis through entropy graph was done to visualize and interpret interaction models identified by MDR. The previously documented main effects of *EPHX1* Tyr113His and *SULT1A1* Arg213His in smokers and CYP1A1*2A and *GSTP1* Ile105Val in non-smokers were evident. Further, synergistic interactions of *SULT1A1* Arg213His with *GSTP1* Ile105Val and with CYP1A1*2C were observed in non-smokers.

As haplotype are more efficient and informative than separate markers, haplotype association analysis was carried out in *CYP1A1* and *EPHX1* genes. CG haplotype in *CYP1A1* was significantly associated with risk of lung cancer. Noteworthy were results in *EPHX1*, where frequency of haplotypes among cases was strikingly similar to report published in esophageal cancer from north India [56].

Although both MDR and CART validated LR results, yet they differed in identifying some unique interactions, reflecting different methods followed by each program. Both approaches provide a clear advantage over the traditional LR by identifying non-linear interactions among discrete genetic and environmental attributes. Significant findings of the study are summarized in Figure S2. It would be safe to assume a definite association of the commonly recognized factors to lung cancer that might have implications on future studies. Role of CYP1A1*2A polymorphism is evident only among non smokers in all the three methods. LR and CART analyses even showed a gene-dosage effect for the increased lung cancer risk with the increasing number of variant allele in the CYP1A1*2A polymorphism. As aforesaid, this finding provides support to previously published reports [37]–[40]. MDR and CART analysis show epitasis between *EPHX1* Tyr113His and *SULT1A1* Arg213His polymorphisms exclusively among smokers. Their combined models confer risk to lung cancer however individually both act as protective factors in smokers only. These factors hold their importance as the SNPs are functionally and biologically relevant and have been implicated in the carcinogenesis process in previous studies on various cancers

Major challenge for the identification of true genetic and interactive effects in a multi-factorial study is simultaneous testing of several hypotheses. The three methods of analysis used in this study address the same research hypotheses but differ in terms of their statistical methodologies and analytical approaches. P-value adjustment for multiple testing was performed through SIDAK correction in LR model with the equality as (1-(1-α)^1/n^) where n = 4 both in total and stratified analyses. Multiple testing in data mining approaches such as CART and MDR sometimes compromises upon the comparative power. When numerous null hypotheses are being tested yielding higher order interacting combinations the inference drawn from a single erroneous rejection is not an appropriate strategy, rather the proportion of erroneous rejection needs to be controlled. This is achieved by estimation of FPRP. These approaches utilize internal cross-validations and permutation testing of p-value reducing the chances of making type I errors. Both MDR and CART apply cross validation of data before selecting the best model however MDR also uses 1000 fold-permutation testing, to validate its results for minimizing the proportion of false-positives due to multiple testing. The cross validation (5–10 fold) dividing the whole data set into different sets of training and testing set prevents over-fitting and artificial accuracy improvement. Permutation test is considered the gold standard for accurate multiple testing correction. Controlling for false discovery rate (FDR) is a more realistic approach than as compared to concerns raised by the multiple hypothesis testing. This is because FDR is the proportion of incorrect rejection among all such rejections. Likewise, the best models derived from MDR on total data set and smokers set in this study showed good reliability as associations remained robust even at low prior probabilities for FPRP testing. CART analysis was able to define genetic associations with fairly good measures. Correct classification of cases and controls in test data set was approximately 63% for both.

There might be some limitations to this study. The sample size of our study was relatively small, however based on the evidences (OR) provided by our research group on association between *GST*s with lung cancer [57], the minimum sample size determined was 176 at 5% level of significance and 90% power. Polymorphisms of *EPHX1* Tyr113His and *SULT1A1* Arg213His in cases showed deviation from HWE. After ruling out false positive associations and genotyping errors perhaps population stratification, could be a reason for this deviation. However, the cases were incident, and thus, the data do not show report or recall bias. Also case-control matching was done in reference to age, gender, and ethnicity, thereby controlling for any confounding effect accounted by these variables.

In conclusion this study highlights that better predictors for lung cancer risk can be obtained through polygenic approaches and exploring gene-environment interactions. The study identified distinct patterns of interaction in smoking and non smoking sub groups. However, the results presented should be treated with caution since this is the first epidemiological evidence identifying the complex relationship between genetic polymorphisms and cancer susceptibility in the studied population. Further studies with large samples in independent populations are warranted to validate the findings of this study.

## Supporting Information

Figure S1 **Interaction entropy graphs (for total data set).** The interaction model describes the percentage of the entropy (information gain) removed by each variable (main effect: represented by nodes) and by each pairwise combination of attributes (interaction effect: represented by connections). Attributes are selected on the basis of MDR results obtained in case of total data set. **Labels:** Smk: smoking, SULT: SULT1A1 Arg213His, Ex3: EPHX1 Tyr113His (EH3), Ex4: EPHX1 His139Arg, SULT.
(TIFF)

Figure S2 **Summarized results for LR, MDR and CART analyses.** Green boxes indicate OR<1. Red boxes indicate OR>1. For MDR and CART significant interactions are shown. LR results should be read individually. Alcohol was excluded as it did not appear significant in any analysis.
(TIF)

Table S1 **Detailed list of xenobiotic genes and polymorphisms analyzed in the study.** *Chromosomal position is based on NCBI Build 36.2 (National Center for Biotechnology Information, Bethesda, MD). ** Base pair ^#^mentioned in text as CYP1A1*2A ^##^mentioned in text as CYP1A1*2C ^$^Not Applicable ^†^internal control for *GSTM1* and *GSTT1* multiplex.
(DOC)

Table S2 **Genotype representation and associations under dominant and recessive model between cases and controls.** ^*^crude odds ratio ^**^odds ratio adjusted for smoking, tobacco chewing, betel quid chewing and alcohol.
(DOC)
