# Supplementary figures and images for: Multiple Analytical Approaches Reveal Distinct Gene-Environment Interactions in Smokers and Non Smokers in Lung Cancer
Source: PLoS One. 2011 Dec 19;6(12):e29431. doi: 10.1371/journal.pone.0029431 (PMC3242784; doi:10.1371/journal.pone.0029431)

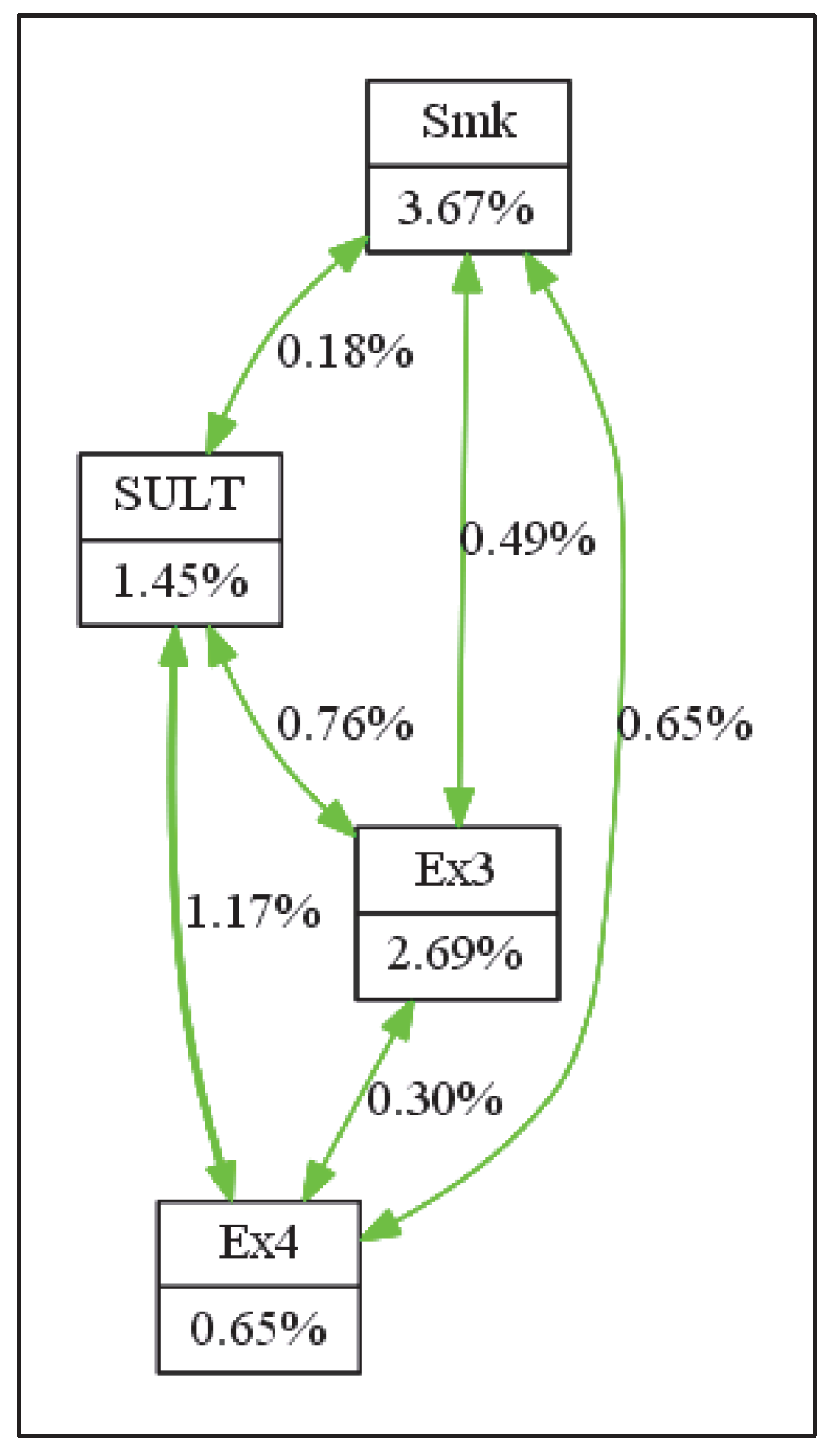

Supplement: Figure S1 — Interaction entropy graphs (for total data set). The interaction model describes the percentage of the entropy (information gain) removed by each variable (main effect: represented by nodes) and by each pairwise combination of attributes (interaction effect: represented by connections). Attributes are selected on the basis of MDR results obtained in case of total data set. Labels: Smk: smoking, SULT: SULT1A1 Arg213His, Ex3: EPHX1 Tyr113His (EH3), Ex4: EPHX1 His139Arg, SULT. (TIFF) [file pone.0029431.s001.tiff]

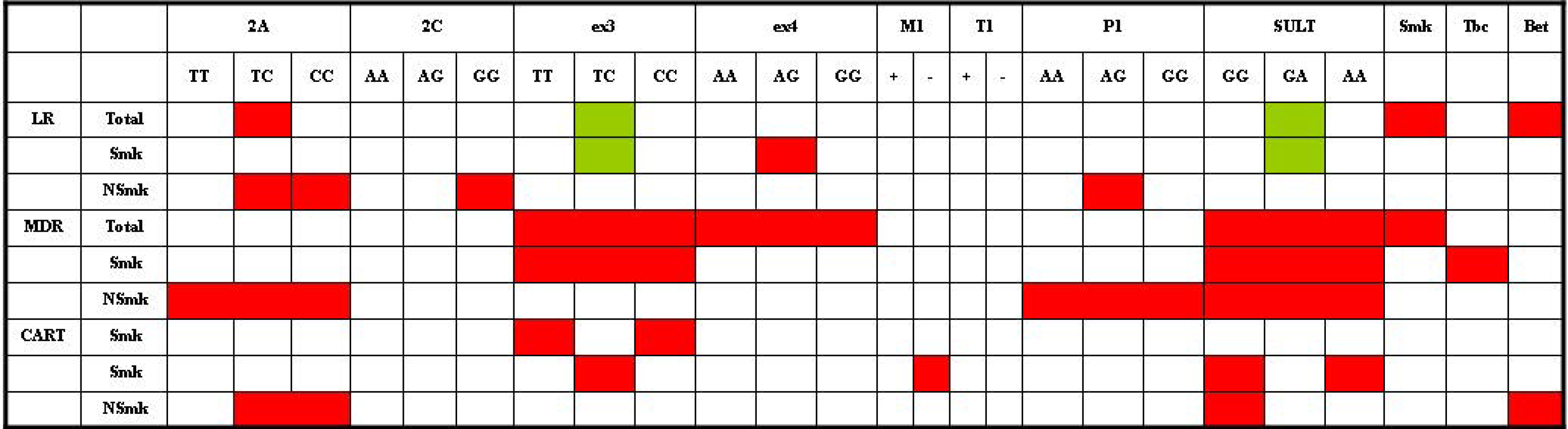

Supplement: Figure S2 — Summarized results for LR, MDR and CART analyses. Green boxes indicate OR<1. Red boxes indicate OR>1. For MDR and CART significant interactions are shown. LR results should be read individually. Alcohol was excluded as it did not appear significant in any analysis. (TIF) [file pone.0029431.s002.tif]
