# Supplementary material for: Multiple Analytical Approaches Reveal Distinct Gene-Environment Interactions in Smokers and Non Smokers in Lung Cancer
Source: PLoS One. 2011 Dec 19;6(12):e29431. doi: 10.1371/journal.pone.0029431 (PMC3242784; doi:10.1371/journal.pone.0029431)
Supplement: Table S1 — Detailed list of xenobiotic genes and polymorphisms analyzed in the study. *Chromosomal position is based on NCBI Build 36.2 (National Center for Biotechnology Information, Bethesda, MD). ** Base pair #mentioned in text as CYP1A1*2A ##mentioned in text as CYP1A1*2C $Not Applicable †internal control for GSTM1 and GSTT1 multiplex. (DOC) [file pone.0029431.s003.doc]

| **Gene** | **Chr*** | **SNP** | **location** | **Polymorphism** | | **Primer sequence** | **bp**** | **Enzyme** | **Ref** |
| --- | --- | --- | --- | --- | --- | --- | --- | --- | --- |
|  |  |  |  | **Nucleotide** | **Codon** |  |  |  |  |
| ***CYP1A1*** | 15q24.1 | rs4646903^#^ | 3’UTR | 6235T>C | NA^$^ | 5'-TAGGAGTCTTGTCTCATGCCT-3'  5'-CAGTGAAGAGGTGTAGCCGCT-3' | 340 | MspI | 9 |
| ***CYP1A1*** | 15q24.1 | rs1048943^##^ | Exon 7 | 2454A>G | Ile462Val | 5'- GAAAGGCTGGGTCCACCCTCT -3'  5'-CCAGGAAGAGAAAGACCTCCCAGCGGGCCA-3' | 333 | NcoI | 12 |
| ***EPHX1*** | 1q42.1 | rs1051740 | Exon 3 | 339T>C | Tyr113His | 5’-GATCGATAAGTTCCGTTTCACC-3’  5’-ATCCTTAGTCTTGAAGTGAGGAT-3’ | 162 | EcoRV | 11 |
| ***EPHX1*** | 1q42.1 | rs2234922 | Exon 4 | 418A>G | His139Arg | 5’-ACATCCACTTCATCCACGT-3’  5'-ATGCCTCTGAGAAGCCAT-3’ | 210 | RsaI | 11 |
| ***GSTM1*** | 1p13.3 | *GSTM1* | Whole  Gene | Presence>Null | Deletion | 5'-GAACTCCCTGAAAAGCTAAAGC-3'  5'- GTTGGGCTCAAATATACGGTGG-3' | 219 | NA | 5 |
| ***GSTT1*** | 22q11.23 | *GSTT1* | Whole  Gene | Presence>Null | Deletion | 5'-TTCCTTACTGGTCCTCACATCTC-3'  5'-TCACCGGATCATGGCCAGCA-3' | 459 | NA | 5 |
| ***GSTP1*** | 11q13 | rs1695 | Exon 5 | 313A>G | Ile105Val | 5’-CCAGTGACTGTGTGTTGATC-3’  5’-CAACCCTGGTGCAGATGCTC-3’ | 189 | BsmA1 | 10 |
| ***β-globin^†^*** | 11p15.5 | NA | NA | NA | NA | 5'-CAACTTCATCCACGTTCACC-3'  5'-GAAGAGCCAAGGACAGGTAC-3' | 268 | NA | 5 |
| ***SULT1A1*** | 16p12.1 | rs9282861 | Exon 7 | 638G>A | Arg213His | 5’-AGTTGGCTCTGCAGGGTTTCT-3’  5’-ACCACGAAGTCCACGGTCTC-3’ | 200 | HhaI | 8 |

**Table S1. Detailed list of xenobiotic genes and polymorphisms analyzed in the study.**

*Chromosomal position is based on NCBI Build 36.2 (National Center for Biotechnology Information, Bethesda, MD).

** Base pair

^#^mentioned in text as *CYP1A1*2A*

^##^mentioned in text as *CYP1A1*2C*

^$^Not Applicable

^†^internal control for *GSTM1* and *GSTT1* multiplex
