# Supplementary material for: Multiple Analytical Approaches Reveal Distinct Gene-Environment Interactions in Smokers and Non Smokers in Lung Cancer
Source: PLoS One. 2011 Dec 19;6(12):e29431. doi: 10.1371/journal.pone.0029431 (PMC3242784; doi:10.1371/journal.pone.0029431)
Supplement: Table S2 — Genotype representation and associations under dominant and recessive model between cases and controls. *crude odds ratio **odds ratio adjusted for smoking, tobacco chewing, betel quid chewing and alcohol. (DOC) [file pone.0029431.s004.doc]

| **Gene** | **Model** | **Effect** | **Reference** | **OR^*^ (95% CI)** | **p value** | **OR^**^ (95% CI)** | **p value** |
| --- | --- | --- | --- | --- | --- | --- | --- |
| ***CYP1A1*2A*** | Dominant | TC+CC | TT | 1.75 (1.66-2.64) | 0.005 | 1.82 (1.20-2.76) | 0.004 |
|  | Recessive | CC | TT+TC | 1.06 (0.62-1.81) | 0.89 | 1.14 (0.66-1.97) | 0.61 |
| ***CYP1A1*2C*** | Dominant | AG+GG | AA | 1.32 (0.87-2.00) | 0.16 | 1.25 (0.82-1.89) | 0.29 |
|  | Recessive | GG | AA+AG | 2.27 (0.78-6.74) | 0.12 | 2.11 (0.74-5.99) | 0.16 |
| ***EPHX1***  **Tyr113His** | Dominant | TC+CC | TT | 0.62 (0.41-0.92) | 0.01 | 0.60 (0.40-0.90) | 0.01 |
|  | Recessive | CC | TT+TC | 1.49 (0.95-2.31) | 0.06 | 1.42 (0.91-2.24) | 0.12 |
| ***EPHX1***  **His139Arg** | Dominant | AG+GG | AA | 1.5 (0.99-2.27) | 0.05 | 1.58 (1.04-2.42) | 0.03 |
|  | Recessive | GG | AA+AG | 1.56 (0.52-4.68) | 0.43 | 2.03 (0.69-5.91) | 0.19 |
| ***GSTP1*** | Dominant | AG+GG | AA | 1.36 (0.92-2.00) | 0.10 | 1.29 (0.86-1.94) | 0.24 |
|  | Recessive | GG | AA+AG | 0.92 (0.36-2.29) | 1.00 | 0.76 (0.30-1.90) | 0.56 |
| ***SULT1A1*** | Dominant | GA+AA | GG | 0.59 (0.39-0.87) | 0.008 | 0.56 (0.37-0.84) | 0.006 |
|  | Recessive | AA | GG+GA | 1.11 (0.52-2.32) | 0.85 | 1.16 (0.56-2.41) | 0.67 |

**Table S2. Genotype representation and associations under dominant and recessive model between cases and controls.**

^*^ crude odds ratio

^**^ odds ratio adjusted for smoking, tobacco chewing, betel quid chewing and alcohol
